# Positron Emission Tomography Scan Reveals an Unusual Source for Cervical Lymphadenopathy: Right Colon Cancer

**DOI:** 10.4103/1319-3767.77249

**Published:** 2011

**Authors:** Silvia Palmisano, Biagio Casagranda, Giuseppe Piccinni, Nicolò de Manzini

**Affiliations:** Department of Surgery, University of Trieste and Cattinara Hospital, Strada di Fiume, Trieste, Italy; Department of Biological Sciences and Human Oncology, Section of General and Oncologic Surgery, University Medical School of Bari, Policlinico, Bari, Italy

**Keywords:** Cervical lymphadenopathy, colon cancer, metastasis, positron emission tomography

## Abstract

Cervical lymphadenopathy is usually related to the presence of an inflammatory disease or to a malignant localization. In the event of metastatic findings, the thyroid gland is the most likely primary site of a tumor. Metastatic lymphadenopathy represents a challenge when the primary tumor is unknown. A 64-year-old female with a supraclavicular palpable mass in the absence of underlying thyroid disease underwent ultrasonography of the neck, biopsy of the pathological lymph node, fluorodeoxyglucose-Positron Emission Tomography (FDG-PET) and colonoscopy until right colon cancer was diagnosed. FDG-PET is a fast and reliable tool to discover the primary site of cervical masses of unknown origin.

Colorectal cancer is the second most common malignancy in developed countries and is the second leading cause of cancer-related death.[1] Metastatic disease from colon cancer is a common event in clinical practice. At the time of diagnosis, 15-20% of patients with colorectal cancer have synchronous liver metastases and an additional 35-45% of patients will develop hepatic metastases during the course of their disease. Up to one-third of patients initially diagnosed with American Joint Committee on Cancer (AJCC) stage I or II develop systemic disease. The liver is the preferred target of colorectal metastases by hematogenous dissemination. Lung, brain and peritoneal carcinomatosis have less incidence of metastatic spread. Symptoms related to the presence of neoplastic lesions of right colon cancer are usually anemia, asthenia, right-side abdominal pain and occult bleeding. Lymphatic spread of tumor cells is an orderly process with initial migration to the regional nodes through tiers of lymph node. Skip metastases are, however, recognized with an incidence of up to 18% when molecular techniques are used to detect the presence of micrometastases.[2] Cervical lymphadenopathy is a very rare site of disease progression from colon cancer, especially in the absence of any other site of metastasis. In a previous report from New York, in a period of 24 years, only three cases of laterocervical metastasis from colorectal cancer were observed over 54,502 cancer patients.[3] Diagnostic work-up to understanding the site of Cancer of Unknown Primary (CUP) can be both time and money consuming and sometimes unsuccessful. CUP is defined as the presence of histologically proven metastatic disease without identification, at the time of diagnosis, of the primary site despite diagnostic work-up. In our opinion fluorodeoxyglucose-Positron Emission Tomography (FDG-PET) could help to quickly resolve the enigma of CUP and to better understand the route map of metastasis.

## CASE REPORT

**Figure 1:**
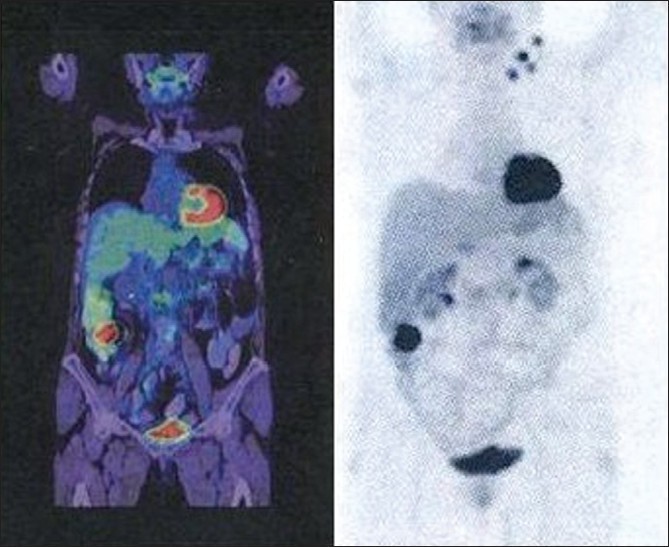
Fluorodeoxyglucose-Positron Emission Tomography. Increased uptake in the correspondence of the right colon (Standardized Uptake Value, SUV = 12.1)

A 64-year-old woman was admitted with an indolent left cervical palpable mass but without dysphagia, dyspnea or voice alteration and a 3-month history of asthenia and anorexia. Physical examination was normal except for the presence of a palpable left supraclavicular mass. No other pathological findings were detected in the chest and abdomen by CT-Scan. Doppler ultrasonography of the neck revealed supraclavear hypoechogenic lymphnodes with predominant peripheral vascularization. A fine needle biopsy was performed. The anatomopathological examination highlighted carcinomatosis cells according to a metastatic site of a glandular type carcinoma. Routine blood test and oncomarkers were normal. Papillary thyroid cancer was suspected, but clinical evaluation and thyroid ultrasonography were normal. In order to reveal the presence of thyroid microcarcinoma a FDG-PET was performed and, unexpectedly, an increased uptake in the right colon was found [Figure 1]. Colonoscopy confirmed an adenocarcinoma in the ascending colon and so a laparoscopic right colon resection was planned. At abdominal exploration, neither liver metastases nor peritoneal carcinomatosis were present. Histology confirmed a pT3N0M1(G2) colonic adenocarcinoma. After 1 year of adjuvant chemotherapy (CAPOX) a cervical ultrasonography showed the regression of the supraclavicular mass and no more disease localizations at the follow-up controls.

## DISCUSSION

Monolateral cervical lymphadenopathy is a common reactive sign of head and neck disease and in the absence of a history of inflammation, a neoplastic cause must be sought. CUP represents a diagnostically challenging disease because it is a metastatic expression of a head and neck cancer. In these cases, the necessity to identify the primary site is in order to provide better targeting therapy. In this view, different instrumental exams are necessary to define the neoplastic development of the cervical lymphadenopathy. A fine needle biopsy of the lymph nodes is a feasible and safe procedure to characterize the type of the unknown primary tumor. A squamous cell carcinoma, or an adenocarcinoma, or a lymphoma or an undifferentiated carcinoma are the most likely histological findings. In our case, an adenocarcinoma was found so the first hypothesis considered by the authors was a thyroid cancer[4,5] then excluded by ultrasonography. At that time, according to Pavlidis,[6] we needed extensive diagnostic work-up to identify the primary site and, as reported in 80% of cases, this protocol is not exhaustive either. In the remaining numbers FDG-PET is able to detect the primary site in about 25% cases. In our experience we decided to quickly identify the primary site of CUP by using FDG-PET. FDG-PET imaging presents sensitivity and specificity rates in lymph node metastasis detection of about 95-100% and 92-94%, respectively,[7,8] and in a recent report from Houston, Texas the overall sensitivity, specificity and accuracy of PET-CT in detecting a colonic lesion were 53%, 93% and 85%, respectively.[9] In this experience PET was able to localize the primary tumor in correspondence of the right colon as subsequently confirmed by pancolonoscopy.[10] Right colon cancer symptoms are usually anemia, asthenia, right-side abdominal pain and occult bleeding but cervical metastatic lymphadenopathy is an unusual localization especially in the absence of any other site of metastasis. Diagnosis of the primary site when challenged with a cervical metastatic lymphadenopathy could be really a difficult and time-consuming process. A metastatic site from colorectal cancer is even more rarely described in literature especially when cancer cells skipped natural filters like the liver and lungs. In the cited recent diagnostic protocol of CUP, panendoscopy is one of the steps necessary to resolve the enigma and whole-body MRI must be included in the patient’s study. Endoscopies are really invasive for the patient and whole-body MRI is as expensive as FDG-PET. With this report we intend to underline the need to submit the patient with CUP to FDG-PET to achieve the final diagnosis, after a failed classical established diagnostic route, without forgetting the possibility of metastasis from colorectal origin. Finally this diagnostic technique, despite its current limits, should not be considered either more invasive or more expensive than other diagnostic tools.
